# Genome Dynamics and Temperature Adaptation During Experimental Evolution of Obligate Intracellular Bacteria

**DOI:** 10.1093/gbe/evad139

**Published:** 2023-07-29

**Authors:** Paul Herrera, Lisa Schuster, Markus Zojer, Hyunsoo Na, Jasmin Schwarz, Florian Wascher, Thomas Kempinger, Andreas Regner, Thomas Rattei, Matthias Horn

**Affiliations:** Centre for Microbiology and Environmental Systems Science, University of Vienna, Vienna, Austria; Centre for Microbiology and Environmental Systems Science, University of Vienna, Vienna, Austria; Centre for Microbiology and Environmental Systems Science, University of Vienna, Vienna, Austria; Centre for Microbiology and Environmental Systems Science, University of Vienna, Vienna, Austria; Centre for Microbiology and Environmental Systems Science, University of Vienna, Vienna, Austria; Centre for Microbiology and Environmental Systems Science, University of Vienna, Vienna, Austria; Centre for Microbiology and Environmental Systems Science, University of Vienna, Vienna, Austria; Centre for Microbiology and Environmental Systems Science, University of Vienna, Vienna, Austria; Centre for Microbiology and Environmental Systems Science, University of Vienna, Vienna, Austria; Centre for Microbiology and Environmental Systems Science, University of Vienna, Vienna, Austria

**Keywords:** host-microbe relationship, environmental chlamydiae, amoeba, evolution experiment, temperature adaptation, genome evolution, symbiont

## Abstract

Evolution experiments with free-living microbes have radically improved our understanding of genome evolution and how microorganisms adapt. Yet there is a paucity of such research focusing on strictly host-associated bacteria, even though they are widespread in nature. Here, we used the *Acanthamoeba* symbiont *Protochlamydia amoebophila*, a distant relative of the human pathogen *Chlamydia trachomatis* and representative of a large group of protist-associated environmental chlamydiae, as a model to study how obligate intracellular symbionts evolve and adapt to elevated temperature, a prerequisite for the pivotal evolutionary leap from protist to endothermic animal hosts. We established 12 replicate populations under two temperatures (20 °C, 30 °C) for 510 bacterial generations (38 months). We then used infectivity assays and pooled whole-genome resequencing to identify any evolved phenotypes and the molecular basis of adaptation in these bacteria. We observed an overall reduction in infectivity of the symbionts evolved at 30 °C, and we identified numerous nonsynonymous mutations and small indels in these symbiont populations, with several variants persisting throughout multiple time points and reaching high frequencies. This suggests that many mutations may have been beneficial and played an adaptive role. Mutated genes within the same temperature regime were more similar than those between temperature regimes. Our results provide insights into the molecular evolution of intracellular bacteria under the constraints of strict host dependance and highly structured populations and suggest that for chlamydial symbionts of protists, temperature adaptation was facilitated through attenuation of symbiont infectivity as a tradeoff to reduce host cell burden.

**Significance**

Bacteria infecting eukaryotic cells have a profound impact on all realms of life and can substantially affect host health and ecosystem functioning. However, we have limited knowledge about evolutionary processes in strictly intracellular bacteria due to their host-dependent lifestyle. Here, we designed an evolution experiment to investigate temperature adaptation in an obligate intracellular amoeba symbiont related to the major human pathogen *Chlamydia trachomatis*. Genome evolution dynamics of the symbiont populations were tracked for over 500 generations, which led to an attenuated infectivity phenotype at elevated temperature, likely ensuring the overall fitness of the microbe-host relationship. These findings have revealed fresh insights into molecular evolution and temperature adaptation of a strictly host-dependent bacterial symbiont.

## Introduction

Nature is replete with bacteria that reside within eukaryotic cells. Examples of intracellular bacteria are present throughout the tree of life, and they are found in virtually all eukaryotes, from protists to fungi, plants, and animals. Intracellular bacteria reside directly in the cytoplasm of the host or host-derived vacuoles (Ray et al. 2009), although exceptions do exist (Schulz and Horn 2015). Host-associated intracellular microbes shape the biology, ecology, and evolution of their hosts in profound ways, affecting processes and functions including development (Braendle et al. 2003) and nutrition (Bäckhed et al. 2005), as well as immunity (Macdonald and Monteleone 2005), pathogen defense (Arthofer et al. 2022; Dharamshi et al. 2022), and speciation (Hurst and Werren 2001).

The intracellular lifestyle of bacteria is ancient (Moran et al. 2008; Subtil et al. 2014). The interplay of microbes with predatory protists provided a “training ground” for the emergence of mechanisms to manipulate eukaryotic hosts, survive phagocytosis, and eventually exploit eukaryotic cells as an ecological niche (Molmeret et al. 2005). The documented ability of many bacterial pathogens of humans to thrive in amoebae and the conservation of mechanisms for interference with phagocytosis in protists and macrophages are evidence of these primordial bacteria-host relationships (Strassmann and Shu 2017). Still, the transition from protists to multicellular eukaryotic hosts is a pivotal evolutionary step, which remains largely unexplored.

One group of bacteria that has made this transition early on during evolution and is now associated with a broad range of eukaryotic hosts is the phylum *Chlamydiae* (a.k.a. *Chlamydiota*) (Collingro et al. 2020). The chlamydiae are well-known for being successful bacterial pathogens of humans, with two members in particular. *Chlamydia pneumoniae* is responsible for as many as 10% of community-acquired pneumonia cases (Grayston et al. 1993; Burillo and Bouza 2010) and has been associated with chronic diseases such as asthma, atherosclerosis as well as Alzheimer's disease (Balin et al. 1998; Campbell and Kuo 2004; Shima et al. 2010)*. Chlamydia trachomatis* comprises more than 15 serologically defined variants and causes over 100 million infections worldwide every year (Wright et al. 2008; Roulis et al. 2013; Dal Conte et al. 2014). Apart from humans, chlamydiae also infect a large variety of animals, including fish, birds, and mammals, causing numerous clinically important diseases in economically significant livestock species such as cattle, goats, sheep, and pigs (DeGraves et al. 2003; Polkinghorne et al. 2009; Schautteet and Vanrompay 2011). But not all chlamydiae are so pathogenic in nature. The so-called environmental chlamydiae are found ubiquitously in the environment (Horn 2008; Collingro et al. 2020) and many of them are considered to be symbionts of free-living amoebae or other protists, with a limited ability to multiply in nonprotozoan hosts (Maurin et al. 2002; Greub et al. 2003; Collingro et al. 2005a; Casson et al. 2006; Kebbi-Beghdadi et al. 2011; Sixt et al. 2012).

Chlamydiae most likely represents one of the most ancient groups of strictly intracellular bacteria. Comparative and phylogenetic genome sequence analysis suggested that the last common ancestor of all extant chlamydiae already resided within a eukaryotic host, very likely an ancient protist, several hundreds of millions of years ago (Horn et al. 2004; Subtil et al. 2014; Taylor-Brown et al. 2015, 2020; Dharamshi et al. 2023). They all share a characteristic biphasic developmental cycle comprising two distinct morphological and physiological stages: the elementary body (EB) survives the extracellular environment and infects new host cells, whereas the reticulate body (RB) replicates inside a host-derived vacuole (Abdelrahman and Belland 2005; Horn 2008). Chlamydiae possess small, reduced genomes and lack essential biosynthetic pathways and therefore rely on numerous metabolites from their eukaryotic hosts (Omsland et al. 2014). Chlamydiae also interact with diverse host cellular pathways and organelles (Elwell et al. 2016; Fischer and Rudel 2018). Owing to the intimate relationships with their host cells, like many strictly intracellular microbes, chlamydiae have so far eluded cultivation in host-free media and are thus challenging to study.

Here we used a combination of evolution experiments with phenotype assays and genome resequencing to investigate host adaptation of chlamydiae. Experimental evolution has been of utmost importance to the field of evolutionary biology, from testing evolutionary theories to studying tradeoffs and constraints to environmental adaptation (Kawecki et al. 2012). Yet, despite the usefulness of this approach and the multitude of studies on free-living microbes, strictly intracellular bacteria have rarely been investigated using evolution experiments due to the challenges inherent to host-dependent systems. The chlamydiae, in particular, those naturally associated with unicellular eukaryotes, therefore provide us with a distinct opportunity to gain an initial understanding of the evolutionary dynamics in obligate intracellular bacteria (Joseph et al. 2012; Borges et al. 2013; Herrera et al. 2020). Here, we have established replicate populations of the environmental chlamydia *Protochlamydia amoebophila* thriving in *Acanthamoeba* castellanii Neff hosts under two temperatures (20 °C, 30 °C) for 510 generations (38 months), and we estimated the spontaneous mutation rate of these symbionts by setting up a mutation accumulation (MA) experiment. We observed a temperature-dependent decreased infectivity of symbionts in the evolved populations and, by tracking genome dynamics, we identified mutations that may have played an adaptive role. Together, these evolution experiments shed new light on the process and molecular basis of temperature adaptation in protist-associated chlamydiae, a crucial step in the transition from protist symbionts to pathogens of humans and animals.

## Results

### The Evolution Experiment

We designed the experiment by making use of two temperatures (20 °C, 30 °C) so as to be able to look at the processes involved in the temperature adaptation of the *Protochlamydia* symbiont in its *Acanthamoeba* host. Starting with an ancestral amoeba culture infected with the strictly intracellular symbionts, the 20 °C treatment served to represent adaptation under normal culture conditions, whereas the 30 °C treatment represented an adaptation to elevated temperature.

The evolution experiment included 12 replicates for each temperature treatment, consisted of regular transfers of infected amoeba host cells to fresh media, and ran for a total of 38 months, approximately 510 bacterial generations (see Materials and Methods section). Phenotype assays and pool sequencing of numerous replicates were performed at different time points throughout the experiment (supplementary fig. S1, Supplementary Material online). The ancestral *P. amoebophila* strain was used as a reference to identify all variants that arose throughout the experiment (supplementary table S1, Supplementary Material online). 61 samples were sequenced in total across six time points. We sequenced every 5–7 months as we predicted that this allowed sufficient time for a few mutations to arise and be selected for in the different treatments. We sequenced a larger number of replicates from the 30 °C treatments (*n* = 37) as we were particularly interested in adaptation to this elevated temperature. We were keen to identify as many variants as possible that were potentially beneficial and therefore increased in frequency in the population. This greater sequencing effort allowed us to identify any cases of parallelism among replicates.

### Temperature-Dependent Attenuation of Infectivity

The fitness of strictly intracellular bacteria is largely determined by their ability to establish and maintain infections in their host cell population. We thus performed infection assays to determine whether the infectivity of the evolved symbionts was different from that of the ancestral population. This was carried out by infecting naive (symbiont-free) ancestral amoebae with EBs isolated from a specific treatment at a particular time point and measuring the mean percent of highly infected amoebae 96 h postinfection (hpi), equivalent to the duration of one full developmental cycle of the symbiont (König et al. 2017). The infection assays were performed at both ambient (20 °C) and elevated (30 °C) temperatures.

Overall we observed that the infectivity remained fairly constant throughout the evolution experiment at either assay temperature for the symbionts isolated from the 20 °C treatment replicates, but the infectivity was higher in assays performed at the ambient temperature from which the symbionts were isolated originally (supplementary fig. S2*A*  and  *B*, Supplementary Material online). Symbionts from the 30 °C treatment replicates displayed lower infectivity at either assay temperature than those symbionts from the 20 °C replicates (supplementary fig. S2, Supplementary Material online). As the experiment progressed, the infectivity of the symbionts from the 30 °C replicates significantly decreased further, especially when the assays were performed at ambient temperature (supplementary fig. S2*C*  and  *D*, Supplementary Material online). This implies that as the experiment progressed, the symbionts evolved at the elevated temperature and developed an attenuated infectivity.

**Fig. 1.**
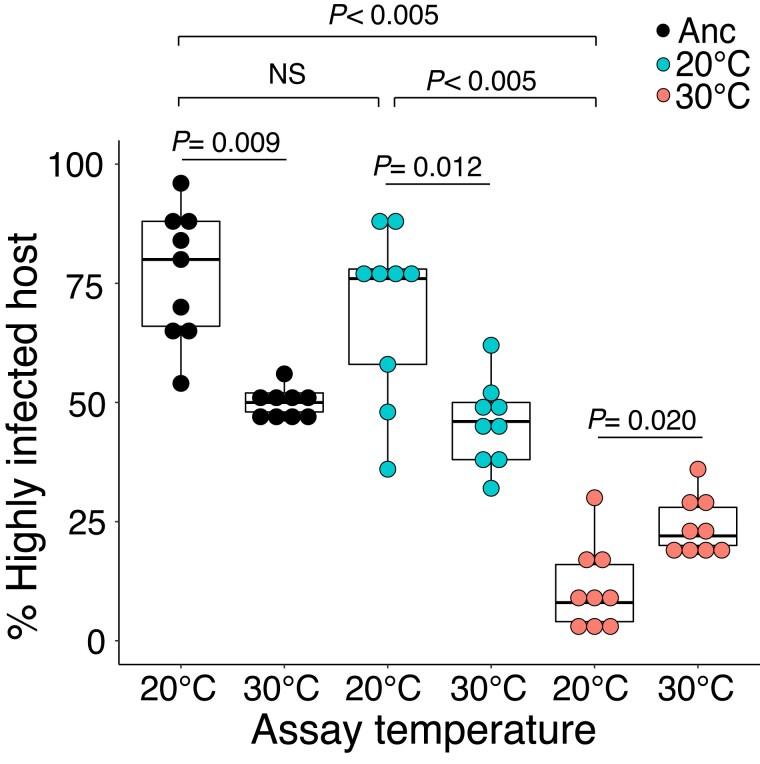
Infectivity of ancestral and evolved symbionts. Naive amoebae (*n* = 9) at a concentration of 10^5^ cells per mL were infected with *P. amoebophila* symbionts isolated from the ancestor and evolved populations after 415 generations with an MOI of 30. The percent of highly infected amoebae was assessed at 96 hpi using FISH. In the box plots, the interquartile range (IQR) between the first and the third quartiles is indicated by the box, while vertical lines extend to a distance of 1.5×IQR from the first or third quartile. The horizontal line within the box represents the median. The infectivity among the different treatments at 20 °C was compared using a Kruskal–Wallis test (χ^2^ = 17.762, *P* < 0.005) followed by Dunn's multiple comparison post-hoc tests. The infectivity between paired samples at different temperatures was compared using a Wilcoxon signed-rank test.

We performed the most comprehensive analysis with symbionts from both treatments obtained after 31 months (415 generations). At ambient temperature, the infectivity of symbionts evolved at 20 °C did not significantly change when compared to ancestral EBs (fig. 1). Confirming our previous analysis, this is not the case with EBs evolved at the higher temperature, where a pronounced and significant decrease in infectivity was measured (fig. 1). When performing the infectivity assays at 30 °C, the infectivity of the symbionts was significantly lower than at 20 °C for the ancestor and the populations that evolved at 20 °C (fig. 1). Although the infectivity of symbionts from the 30 °C treatment was the lowest at this temperature, it was still significantly higher than the infectivity of those same symbionts when the assays were performed at 20 °C (fig. 1). This suggests that the chlamydiae maintained for a long period at the elevated temperature did, in fact, adapt to these environmental conditions.

Since these experiments were carried out using ancestral amoeba host cells that were maintained in the laboratory environment at 20 °C, we wanted to account for this potential bias relating to the host used. To this end, we also carried out a subset of the infectivity assays using uninfected amoebae that had evolved at 30 °C for the duration of the experiment in order to investigate the host effect on infectivity. There was no statistically significant difference in infectivity for any of the symbionts when performing the infectivity assays using amoebae adapted to the elevated temperature (supplementary fig. S3, Supplementary Material online). We also cured the amoebae from two replicates in the 30 °C treatment from their chlamydial symbionts using rifampicin. These symbiont-free amoebae were then re-infected, at both 20 °C and 30 °C, with co-evolved EBs that had been isolated before the curing procedure. Once again, there was no change in infectivity for these 30 °C evolved EBs when re-infecting their co-evolved hosts, demonstrating that the reduction in infectivity was not due to the introduction to a novel host, but was a function of the symbionts themselves (supplementary fig. S4, Supplementary Material online). Finally, in order to assess the stability of this loss of infectivity, we took populations from three replicates in the 30 °C treatment and maintained them at 20 °C for 8 weeks, equivalent to around 27 generations. After performing infectivity assays, it was evident that there was no significant increase in infectivity of these EBs at the lower temperature as compared to their infectivity after isolation from the 30 °C treatment (data not shown). This implied that the loss of infectivity in the 30 °C populations was a stable phenotype.

### Dynamics of Genome Evolution

Next, we performed pooled whole genome resequencing of in total 61 symbiont populations from diverse time points and replicates (supplementary fig. S1, Supplementary Material online). We identified all the variants that arose throughout the evolution experiment in all the replicates across both treatments, and this amounted to 282 mutations (supplementary table S1, Supplementary Material online). The majority of the variants were single nucleotide polymorphisms (SNPs, 79.1%) as opposed to small insertions/deletions (indels <4 nt, 20.9%), and from these SNPs, a much larger proportion was identified as being in the protein-coding region (82.5%) compared to the intergenic region (17.5%), although these values did not deviate from a random distribution. Most of the SNPs in the protein-coding region were nonsynonymous mutations (80.4%), with nearly a fifth of the SNPs (19.6%) being synonymous and not causing any alteration in the encoded amino acid.

**Fig. 2.**
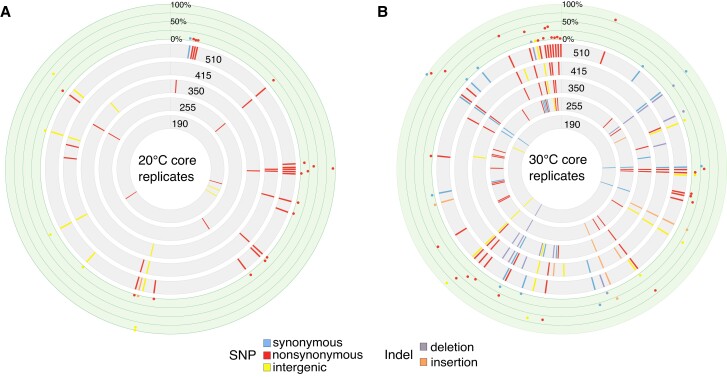
Emergence of mutations across the symbiont genome throughout 510 generations. Variants in the *P. amoebophila* genome during long-term evolution in the core replicates at (*A*) ambient and (*B*) elevated temperature are shown as circular plots. The five gray rings, from inner to outer, show mutations present in genomes sampled at 190, 255, 350, 415, and 510 generations. SNP mutations are separated according to whether they are nonsynonymous (red), synonymous (blue), or intergenic (yellow). Indels are separated according to whether they are deletions (purple) or insertions (orange). The frequencies of the mutations present at 510 generations are shown in the outermost green ring. Genome visualization was carried out with Circos (Krzywinski et al. 2009). Molecular details for all mutations are shown in supplementary table S1, Supplementary Material online.

Three replicates from each temperature were sequenced at every time point from 190 generations onward (supplementary fig. S1, Supplementary Material online). These are referred to here as “core replicates” and allow for more comprehensive analyses of evolutionary changes during the experiment. In total we identified 123 SNPs and 19 small indels across all six core replicates. No apparent mutational hotspots were observed across the 2.41 MB genome of *P. amoebophila* (fig. 2). Fewer mutations (*n* = 37) were identified in the core replicates at ambient temperature and the majority of these were SNPs, with only one indel present (fig. 2*A*). Twenty-six of these SNPs were nonsynonymous mutations and four intergenic mutations were in potential promoter regions. The core replicates at the elevated temperature had more than double the number of SNPs (*n* = 87) than those from ambient temperature, with 18 indels being identified at 30 °C (fig. 2*B*). Fifty-one of these SNPs were nonsynonymous mutations and ten intergenic mutations were in potential promoter regions. After 510 generations, a majority of the variants identified in the 20 °C replicates were low frequency as opposed to those identified in the 30 °C replicates (fig. 2).

The ambient temperature should not have been stressful for either host or symbiont as 20 °C represents standard laboratory conditions used for culture maintenance. The elevated temperature was expected to create a more challenging environment for the chlamydial symbionts as both host and bacteria are used to thriving at ambient temperatures in their natural environment, well below 30 °C. The higher occurrence of nonsynonymous SNPs, small indels (<4 nt), and mutations in potential promoter regions at 30 °C suggests that many of these variants were beneficial and allowed the symbionts to adapt to the elevated temperature.

We noted that one of the 30 °C replicates (replicate 4; supplementary table S1, Supplementary Material online) evolved a moderately elevated mutation phenotype at some point between 190 and 255 generations. At its first sequenced time point (190 generations) only two mutations in total had been identified. However, the number of mutations (SNPs and small indels <4 nt) identified in this replicate increased around 4-fold at the next sequenced time point and remained elevated when compared to the other replicates of the same treatment throughout the remainder of the experiment (table 1). Five variants were identified in this replicate that arose by 255 generations and subsequently remained in the population throughout the course of the evolution experiment (table 1). The observed elevated number of mutations in this symbiont population could be the result of a nonsynonymous SNP located in the *ung* gene that codes for the enzyme uracil-DNA glycosylase. This enzyme catalyzes the first step in a repair pathway for uracil-containing DNA in *Escherichia coli* and in many other organisms (Lindahl 1979), and *E. coli ung* mutants have previously been shown to display elevated mutation rates (Duncan and Weiss 1982).

**Table 1:** Evolution of an Elevated Mutation Phenotype

Variant Type	Variant Effect	Gene Name	RefSeq Description	255 Gen (%)	350 Gen (%)	415 Gen (%)	510 Gen (%)
SNP	Nonsynonymous	NA	Hypothetical protein	13.7	28.7	39.0	34.9
SNP	Nonsynonymous	rpsA	30S ribosomal protein S1	27.0	74.8	59.4	66.1
SNP	Nonsynonymous	rumA	RNA methyltransferase	19.0	24.7	38.7	37.5
SNP	Nonsynonymous	ung	Uracil-DNA glycosylase	6.4	23.0	42.0	37.4
INS	Frameshift	NA	Hypothetical protein	3.3	20.3	25.2	30.6
Number of mutations			Mean of other replicates	5.6	6.5	10.5	9.3
			Replicate 4	24	19	24	35
			Fold increase	4.3	2.9	2.3	3.8

The persistent variants and the elevated number of mutations in replicate 4 of the 30 °C treatment are shown.

### Adaptive and Persistent Mutations

**Fig. 3.**
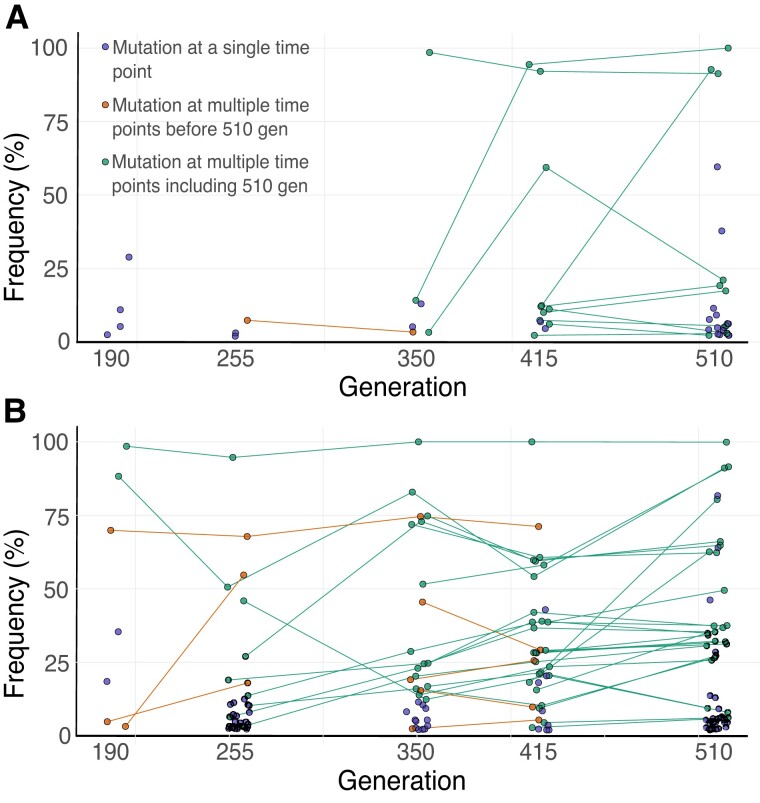
Mutational trajectories of symbiont populations at ambient and elevated temperature. Trajectories of novel mutations throughout the experiment in the core replicates at (*A*) 20 °C and (*B*) 30 °C are shown. Lines connect the same mutation over multiple points. Mutations that appear once are shown in purple. Mutations that persist over multiple time points are shown in orange if they are not present after 510 generations, and in green if they are present after 510 generations. Following all the novel mutations that arose in the core replicates, we observe that at 30 °C (i) more mutations are present; (ii) mutations are more likely to persist over multiple time points; and (iii) mutations tend to reach higher frequencies.

We next analyzed the trajectories of mutations over sequential time points in the core replicates, which should shed light on the fitness effects of the mutations observed (fig. 3). It is evident that the 30 °C replicates accumulated more mutations throughout the experiment compared to the ambient temperature. More importantly, we could identify those mutations that persisted over multiple time points, which would indicate that they likely provided a benefit as they were being maintained in the population. In fact, by the final time point, the amount of mutations identified at 30 °C that had already been present earlier in the experiment (*n* = 26) was more than double the amount at 20 °C (*n* = 10). Nine mutations were already present in the 30 °C treatment replicates from 255 generations, whereas none of the mutations in the 20 °C treatment replicates persisted for this long. At 20 °C, mutations tended to arise at one time point and were subsequently replaced by mutations at the next time point, therefore not really providing any long-term benefit. Finally, overall, mutations reached higher frequencies at the elevated temperature with a median frequency of 20% at the final sequenced time point, as opposed to 6% at the ambient temperature. After 510 generations only eight mutations had a frequency above 15% at 20 °C, with three of those reaching frequencies above 60%. Twenty-six mutations had a frequency above 15% after 510 generations at 30 °C, with ten of those above 60%. This suggests that a number of beneficial mutations were present in the evolved symbiont populations at elevated temperature.

**Fig. 4.**
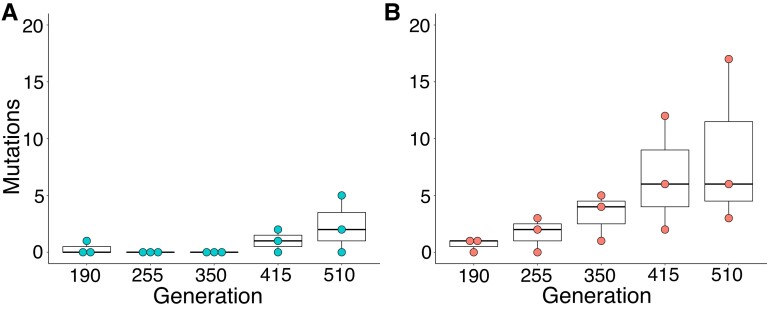
Temperature-dependent differences in the number of persistent and likely adaptive mutations. The number of persistent mutations (SNPs and small indels with a frequency >5%) throughout the experiment for core replicates at (*A*) 20 °C; and (*B*) 30 °C is shown. Whereas the number of persistent mutations in the replicates at 20 °C remained low at all the time points, persistent mutations steadily increased at each time point in the replicates at 30 °C (*P* < 0.05, Mann–Kendall test).

Since the novel mutations that persisted are generally expected to provide an adaptive advantage, we took a closer quantitative look at the differences in the number of persistent mutations in the core replicates—here defined as mutations (SNPs and small indels <4 nt) identified in subsequent time points at a frequency of at least 5% (fig. 4 and supplementary table S1, Supplementary Material online). Few persistent mutations were observed at both temperatures for the first 190 generations. However, as the experiment progressed, those mutations increased in number. This was particularly prevalent at 30 °C, where by the last time point the mean number of persistent mutations was nearly four times greater than the mean number at 20 °C (8.67 vs. 2.33). This confirmed our previous observation (fig. 3) that mutations at the elevated temperature were more likely to persist over multiple time points. In fact, there was a statistically significant increasing trend of persistent mutations over time at 30 °C (*P* < 0.05, Mann–Kendall test) that was not seen at 20 °C (fig. 4).

### Convergence and Divergence Among Replicate Populations

Our experimental set-up also allowed us to study the specificity of genomic evolution which is the extent of parallel evolution during temperature adaptation of the symbiont populations. To this end, we compared mutations identified at the final sequenced time point across all replicates from both treatments, among replicates within the same treatment and between the two treatments. We only included those mutations that solely impacted one single gene, termed “qualifying” mutations (Deatherage et al. 2017) (see Materials and Methods). A total of 98 qualifying mutations were identified to fit our criteria for the analyses. Dice's Coefficient of Similarity, *S*, was then computed for each pair of evolved replicate populations.

Across all replicates from the two treatments, the grand mean similarity, *S*_m_, is 0.04, which is not statistically significant. The mean within-treatment similarity, *S*_w_, is 0.06 (*P* < 0.01, F-test) and 0.05 (*P* < 0.01, F-test) at 20 °C and 30 °C, respectively. Both these values are significant, indicating that the mutated gene overlap between replicates within the same treatment is greater than expected by chance. The mean between-treatment similarity, *S*_b_, between these two treatments is 0.02, which is not statistically significant. Replicates from the same treatment were therefore more similar to each other with respect to their mutated genes than replicates from a different treatment, indicating an adaptation to their particular experimental conditions. Sixteen genes were affected by at least two qualifying mutations across the two treatments (supplementary table S2, Supplementary Material online). Six genes carried mutations at both temperatures, whereas ten genes were affected by mutations exclusively at 30 °C, the most prominent gene being identified in five different replicates (PC_RS06415). No genes were solely affected by qualifying mutations across multiple replicates at the lower temperature.

We next investigated which gene functions were affected by mutations in our experiment and thus putatively associated with temperature adaptation. We, therefore, separated all the mutations identified in the final sequenced time point across both temperatures into functional groups based on the eggNOG (v5.0) database (Huerta-Cepas et al. 2019). However, owing to the low mean number of mutations per replicate, we were not able to make any robust conclusions (supplementary fig. S5, Supplementary Material online and supplementary table S3, Supplementary Material online).

### Spontaneous Mutation Rate

We estimated the spontaneous mutation rate of the *P. amoebophila* symbiont using two methods, which resulted in an upper bound value and a much more conservative value (see Materials and Methods section; table 2). We chose to do this since we studied populations of individuals in our system and not single clones; therefore, these two estimates should provide the range in which the actual spontaneous mutation rate lies. We made use of three replicates that evolved at 20 °C with a monthly 1,000-fold dilution (instead of the 10-fold dilution used in the main experiment), which resembles an MA experiment in which all mutations should accumulate at the rates at which they happen, regardless of fitness effects, with the exception of lethal and highly deleterious mutations (Barrick and Lenski 2013). This resulted in an estimated spontaneous mutation rate between 1.11 × 10^−8^ and 9.76 × 10^−10^ mutations per nucleotide per generation (table 2 and supplementary table S1, Supplementary Material online).

**Table 2:** Estimated Spontaneous Mutation Rates of *Protochlamydia amoebophila*

Method	No. of SNPs	No. of Indels^a^	No. of Replicates	Mutations Per Line	Mutation Rate Per Nucleotide (×10^−8^)^b,c^	Std Dev (×10^−9^)	Mutation Rate Per Genome (×10^−2^)^c^	Std Dev (×10^−2^)
Upper bound	33	1	3	11.33	1.106	8.417	2.698	2.053
Conservative	3	0	3	1.00	0.098	0	0.238	0

a Insertion or deletion of <4 nt.

b Genome = 2,438,912 nt.

c Generations per replicate = 420 gen.

Similarly, we used these same methods to estimate the mutation rate of our symbiont that had evolved at 30 °C with an identical monthly 1,000-fold dilution. The mean number of mutations per line was 11.67 for the upper bound method and two mutations for the conservative method, as calculated using three replicates (supplementary table S1, Supplementary Material online). This produced an estimated mutation rate between 1.14 × 10^−8^ and 1.95 × 10^−9^ mutations per nucleotide per generation. We cannot exclude the effect that the elevated temperature may have had on the symbionts in these three replicates. Nevertheless, the estimated range of the mutation rate between the two temperatures is very similar, suggesting that temperature did not have much influence on this parameter.

## Discussion

Our evolution experiment using an obligate intracellular symbiont of amoeba was designed to provide insights into basic features of molecular evolution and temperature adaptation in strictly host-dependent microbes. Replicate *P. amoebophila* populations evolved for 510 generations in their natural *Acanthamoeba* hosts under two different temperatures. We compared genomic adaptation across both treatments and also determined whether any phenotypic changes occurred throughout the experiment, in particular, to identify how the bacterial symbionts adapted to survive at the higher temperature of 30 °C. This elevated temperature condition provides a source of environmental stress for the symbiont and the host since they naturally dwell at lower temperatures in the environment.

### Attenuated Infectivity Maintains Fitness of Microbe-Host Association at Elevated Temperature

We measured the infectivity of the symbionts as a proxy for bacterial fitness and observed that the infectivity remained fairly constant throughout the evolution experiment for the symbionts from the ambient temperature treatment (fig. 1 and supplementary fig. S2*A*  and  *B*, Supplementary material online). In contrast, symbionts from the elevated temperature treatment displayed increasingly lower infectivity as the evolution experiment progressed (fig. 1 and supplementary fig. S2*C*  and  *D*, Supplementary Material online). Importantly, near the end of the observation period the infectivity of the 30 °C evolved symbionts was significantly higher when the assays were performed at the elevated temperature rather than at 20 °C (fig. 1). Therefore, although the symbionts isolated from the 30 °C treatment developed an attenuated infectivity at either assay temperature as the experiment progressed, these chlamydiae did in fact adapt to the elevated temperature.

Overall temperature adaptation of the symbionts was facilitated through attenuated infectivity. One potential scenario for this is that this phenotype evolved as a tradeoff to reduce host cell burden. We did notice that a proportion of *Acanthamoeba* cells in the cultures maintained at the higher temperature were not as highly infected as amoeba maintained at 20 °C (data not shown). It is thus conceivable that at the elevated temperature, there is a lower maximum bacterial load of the *Acanthamoeba* host cells. Therefore, the reduction in infectivity of the 30 °C evolved symbionts would have been a tradeoff to reduce host cell stress and compensate for the lower maximum bacterial load per amoeba at the elevated temperature. Ultimately, this would have been beneficial as it would have stabilized the fitness of the host-symbiont relationship. Similar findings have been shown in other endosymbiotic bacteria infecting drosophilid flies, relating to the male-killing phenotype (Hurst et al. 2000; Anbutsu et al. 2008). This trait is characterized by the production of all, or nearly all, female broods related to low egg hatch rates, and is caused by *Wolbachia* in *Drosophila bifasciata* females. Parents exposed to elevated temperature resulted in a decrease in bacterial density in eggs as well as a decline in penetrance of the trait, which suggested that a threshold density of *Wolbachia* within embryos is required to result in the male-killing phenotype (Hurst et al. 2000). Another investigation on the effect of high temperature through successive host generations on native and nonnative *Drosophila* host species revealed a reduction in the male-killing phenotype in both host species, but a reduction in infection with the symbiont *Spiroplasma* only in the nonnative Drosophila host (Anbutsu et al. 2008).

### Increased Genetic Diversity Due to Structured Populations of Host-Dependent Microbes

Pooled whole genome resequencing served to study evolutionary processes during our experiment at the molecular level, with the majority of mutations identified across both treatments being SNPs located in protein-coding regions. Overall, mutations in the core replicates increased as the evolution experiment progressed, with a greater number of mutations being identified later in the evolution experiment (see supplementary table S1, Supplementary Material online). This may seem unexpected at first glance as many microbial experimental evolution studies in which the timing of the fixation of adaptive mutations was approximated found a high number of mutations in the early stages of evolution (e.g., Conrad et al. 2009; Lee and Palsson 2010). However, this observation might be a consequence of the obligate intracellular lifestyle and the developmental cycle of the symbionts.

First, bacterial and host cell cycles are tightly linked. The symbionts have a mixed transmission mode (Herrera et al. 2020) but are predominantly transmitted vertically during host cell replication and are thus dependent on binary fission of their amoeba host cells (see Materials and Methods section). As the host cell cycle is slow, the 38 months over which our evolution experiment was carried out only equated to 510 generations. To put this into perspective, an evolution experiment with *E. coli* over 60 days comprised around 1,100 generations (Fong et al. 2005). Our evolution experiment is therefore very much in its infancy where selection is still strong and saturation with new mutations may not have occurred yet.

Second, the strictly host-dependent nature and the primary mode of vertical transmission of our symbionts give rise to a structured environment. The bacterial populations are divided into many subpopulations—within single amoebae—whose fate is each also dependent on individual host fitness. This could result in the maintenance of increased genetic diversity. In fact, the fixation time required for a mutation increases in structured populations and this, in turn, will increase the chance that another beneficial mutation will appear and compete with earlier ones (Gordo and Campos 2006). This pronounced competition via clonal interference (Gerrish and Lenski 1998) leads to a higher number of segregating mutations in the population, that is higher genetic diversity. An increased variation in structured populations has previously been observed in experiments carried out on free-living bacteria such as *Ralstonia* sp., *Pseudomonas fluorescens,* or *E. coli* when comparing populations evolved in structured and homogeneous habitats (Korona et al. 1994; Rainey and Travisano 1998; Habets et al. 2006). In our structured host-microbe system, a mutation that arises during RB replication must compete with other RBs inside the host and eventually needs to be released as an EB. The EB carrying the mutation must then compete against other EBs to enter a new host and finally outcompete the chlamydiae already residing within that new host. This creates multiple levels of selection, and an adaptive advantage at one stage may face a tradeoff by being at a disadvantage elsewhere. Furthermore, the different subpopulations will each fix beneficial mutations conferring small benefits since they do not have access to the rare beneficial mutations of large effect like in larger, well-mixed populations (Orr 1998; Burch and Chao 1999; Miralles et al. 1999). As a consequence, the symbiont populations would have few mutations that actually become fixed, with low-frequency mutations co-existing simultaneously being more common (fig. 3).

A potential limitation of our protocol may arise from the fact that sequencing was carried out on extracellular EBs alone. Although the EBs themselves do represent the RB population, there may have been mutations in the intracellular population that we didn’t see since we used the extracellular EBs as a proxy. The EB is the survival and dispersal stage, and any long-term adaptation in a natural setting would need to go through this stage. However, by following our protocol we could confirm that we only sequenced EBs, whereas the intracellular fraction would have had a mixture of EBs and RBs. Nevertheless, one should keep in mind that the EB stage does represent another level of selection and that there may be more (unseen) genetic variability in the intracellular bacterial population.

The most well-known study in microbial experimental evolution is the famous long-term evolution experiment (LTEE) set up in 1988 by Richard Lenksi to test the repeatability of evolutionary dynamics across 12 replicate *E. coli* populations. Although most of the analysis in the LTEE was carried out after sequencing individual clones, mutational variants in mixed bacterial population samples were also analyzed (Barrick and Lenski 2009). We can roughly compare the average number of SNPs identified at the final sequenced time point (510 gen) of our symbiont populations with the first sequenced time point (2,000 gen) in the *E. coli* LTEE. They identified seven substitutions in total, whereas we calculated that our final populations at 20 °C and 30 °C had an average of 5.3 and 9.1 substitutions per replicate (see supplementary table S1, Supplementary Material online), respectively. If we were to assume that the mutation rate remains roughly the same and extrapolate the number of substitutions to 2,000 generations, it would appear that the symbiont populations at 20 °C and 30 °C have a 3-fold and 5-fold increase in the number of mutations relative to the LTEE, respectively. This elevated number of mutations in our symbiont populations may very well be a result of the increased structure of our host-dependent system relative to the environmentally homogeneous habitat provided in the LTEE (Barrick and Lenski 2009), where mutations can outcompete each other more easily.

### Adaptive Evolution at Elevated Temperature

Whereas synonymous mutations are generally assumed to have no effect on fitness (but see Agashe et al. 2016; Lebeuf-Taylor et al. 2019), nonsynonymous mutations are typically harmful, are selected against, and will be eliminated by purifying selection (Jia et al. 2003). In those rare cases when nonsynonymous mutations confer a selective advantage, they are favored and maintained by positive selection. We identified a higher number of variants in the elevated temperature treatment that were nonsynonymous SNPs, small indels, or else mutations in potential promoter regions. Such mutations alter the protein-coding sequence and affect transcription levels, which suggests that they have allowed the symbionts to adapt to the 30 °C environment. Furthermore, by following the trajectories of the novel variants that arose over time in the core replicates (fig. 3), we observed that numerous variants at 30 °C persisted throughout the experiment until the final time point (*n* = 26). The mean number of persistent variants above 5% at 30 °C is also nearly four times greater than at 20 °C by the last sequenced time point, and we identified a statistically significant increasing trend of persistent variants over time only at 30 °C (fig. 4, *P* < 0.05, Mann–Kendall test). Such mutations were likely adaptive and beneficial, to different degrees, and allowed the symbionts to improve their fitness and fine-tune the host interaction at the elevated temperature.

To study whether there are any mutational hotspots in particular genes or pathways, we first focused on qualifying mutations (Deatherage et al. 2017) in the experiment to calculate Dice's Coefficient of Similarity. This allowed us to compare the similarity of mutated genes within the same treatment and between them, which should provide a good indication as to whether mutations in certain genes or gene pathways are more predominant in one treatment over another. A previous study examined genomic evolution in 30 *E. coli* populations adapting to five different temperature regimes for 2,000 generations (Deatherage et al. 2017). They found that within the same thermal regime clones shared nearly 17% of their mutated genes, while clones evolving under different temperature treatments shared less than 5% of their mutated genes. In our study, we observed a statistically significant similarity of 5–6% across replicates at each temperature, respectively, and a similarity between the temperature regimes of 2%. Replicates from the same treatment thus shared more mutated genes amongst each other than replicates from a different treatment, indicating adaptation to the particular experimental conditions. This confirms temperature as an effective selection pressure and further supports that many of the mutations identified are adaptive. The overall lower mutation specificity in our experiment compared to the *E. coli* experiments is consistent with the observed increased genetic diversity due to the structured symbiont populations. In addition, the longer time frame in the previous study (Deatherage et al. 2017) may have led to more pronounced parallelism. Similar calculations were also carried out using the populations from the *E. coli* LTEE (Lenski 2017). After 500 generations the populations shared 15% of affected genes but this rose to 33% at 2,000 and 5,000 generations, which demonstrates that parallelism increased after the first 500 generations.

In total 16 genes were affected by at least two qualifying mutations across the different treatments and of these, ten genes were restricted to mutations in the 30 °C treatment (supplementary table S2, Supplementary Material online). Three of these genes encode hypothetical proteins, and three others are predicted to be involved in translation, ribosomal structure, and DNA repair. The most prominent of these ten genes was PC_RS06415 (pc1333) because mutations in this gene were identified in five different replicates. PC_RS06415 is similar to known ABC transporter adenosine triphosphate-binding proteins and classified in eggNOG in the category of defense mechanisms. While its exact function remains unknown, homologs are interestingly found in related chlamydial organisms and other intracellular bacteria, such as *Legionella* species. An additional gene in this subset includes *ptsI*, which is predicted to be involved in the phosphotransferase system (PTS), which is incomplete in *P. amoebophila* and likely serves regulatory functions. Two predicted kinases, *phoR,* and *pknD*, are also affected by qualifying mutations in multiple replicates. The Pho regulon is linked to attenuated virulence and alteration of virulence traits in *E. coli* (Crépin et al. 2011) and to host adaptation in *Mycobacterium tuberculosis* (Chiner-Oms et al. 2019). PknD is well conserved among chlamydiae; its inhibition leads to smaller inclusions and less infective progeny in *C. pneumoniae* (Johnson et al. 2009). Together, qualifying mutations occurred in a number of genes involved in sensing, regulation, and host interaction, suggesting they are involved in the adaptive processes observed.

Altogether, the large number of diverse genes and cellular functions affected suggests that adaptation to the elevated temperature was manifold and comprised changes in regulatory pathways, host interaction including metabolite exchange as well as stress response and DNA repair. Yet, although we had observed an overall attenuated infectivity phenotype in the populations evolved at 30 °C (fig. 1 and supplementary fig. S2, Supplementary Material online), we could not identify any mutations in specific genes that are present in a majority of these populations and that could easily explain the reduced infectivity. This might be due to the complex developmental cycle of the symbionts, where changes at various steps of the infection process—during uptake, during establishment of the intracellular niche, during replication, or during secondary differentiation—may fine-tune host interaction and result in a similar outcome, a less infectious phenotype.

### Spontaneous Mutation Rate Consistent With Previous Values

The 1,000-fold dilution populations at ambient temperature served as an MA experiment, and we used this minimal selection condition to estimate the spontaneous mutation rate of these protist symbionts by employing two methods (see Materials and Methods; table 2). The typical rate of spontaneous mutations obtained from such experiments with bacteria is of the order of 10^−10^ to 10^−9^ per nucleotide per generation (Lind and Andersson 2008; Lee et al. 2012; Sung et al. 2012). The rate calculated by Drake for microbes is 3–4 × 10^−3^ per genome per generation (Drake 1991, 2009). With 1.1 × 10^−8^ mutations per nucleotide per generation and 2.7 × 10^−2^ mutations per genome per generation, our upper bound estimate represents an overestimate of the actual mutation rate (table 2), at least one order of magnitude greater than the previous estimates on bacteria. This was expected since we included every single mutation identified throughout the experiment in our calculations (supplementary table S1, Supplementary Material online). Our more conservative estimate of 9.8 × 10^−10^ mutations per nucleotide per generation and 2.4 × 10^−3^ mutations per genome per generation, however, falls within the range determined for other bacteria (table 2). For this estimate, we only considered mutations at 420 generations whose frequency in the population was at least 50% and, thus, present in the major clone of that specific population (supplementary table S1, Supplementary Material online). The more conservative value lies on the lower end of the previously calculated mutation rates and, in fact, the substitution rate of beneficial mutations is expected to be lower in a structured environment relative to a homogeneous population, where purging via selective sweeps should be a relatively rapid process (Gordo and Campos 2006). Overall, our estimate of the substitution rate for the chlamydial symbiont *P. amoebophila* is consistent with values inferred from long-term laboratory propagation of *C. trachomatis* (Borges et al. 2013) or calculated using a phylogenetic approach for *C. trachomatis* and other bacteria (Hadfield et al. 2017).

## Conclusions

Adaptation to elevated temperature is a critical step in the evolution of environmental microbes to pathogens of humans. Here, we investigated how obligate intracellular symbionts of amoebae responded to increased temperature in an evolution experiment spanning 510 bacterial generations. We found that an increase of incubation temperature from environmental conditions to 30 °C selected for phenotypes with reduced infectivity in replicate symbiont populations, likely caused by the necessity to balance host-interaction in this challenging environment. This phenotype coincided with numerous mutations in genes involved in diverse cellular functions. Furthermore, the increase in the number of nonsynonymous mutations, small indels, and mutations in potential promoter regions indicate the adaptive role these variants may have played at this elevated temperature.

There is a striking paucity of long-term evolution experiments with strictly intracellular bacteria, perhaps because of several challenges inherent to studying these systems. This includes experimental limitations, such as the difficulty to establish clonal cultures and the challenges to interpret pool sequencing data, as well as the lack of routine genetic methods for many strictly intracellular bacteria and their protist hosts. In addition, biological features of intracellular microbial symbioses need to be considered, for instance, the coupling of symbiont and host generation times in vertically transmitted symbionts, structured populations as a result of subpopulations being restricted to the intracellular compartment, different levels of selection during the infection process, and the effects of co-evolution with the host. Yet, evolution experiments bear a great potential for improving our knowledge of the evolution of strictly intracellular microbes and microbe-host interaction. As such, the present study uncovered basic features of molecular evolution and temperature adaptation of chlamydial symbionts in their protected intracellular niche, contributing to a better understanding of how these bacteria evolve and may have transitioned from symbionts in microbial eukaryotes in the environment to well-adapted and highly successful pathogens of humans.

## Materials and Methods

### Evolution Experiment

The evolution experiment was founded when ancestral *A. castellanii* Neff was freshly infected with *P. amoebophila* UWE25 (Collingro et al. 2005b). The term “ancestral” is used to refer to the *A. castellanii* Neff amoeba population that was being propagated in the laboratory before the evolution experiment. After a continuous culture was established, the experiment was set up in 24-well plates (Thermo Fisher Scientific Inc., Waltham, MA, USA) under two temperatures (ambient: 20 °C, elevated: 30 °C). Each treatment had 12 replicates with 1 mL trypticase soy yeast extract (TSY) medium (30 g/L trypticase soy broth, Oxoid, 10 g/L yeast extract; pH 7.3) in each well. Populations were propagated for 38 months by weekly 10-fold dilutions in 1 mL TSY (allowing for ∼3.35 generations per week). Samples were taken every 2–3 months and stored with 10% (v/v) dimethyl sulfoxide (DMSO) as a cryoprotectant in liquid nitrogen, where they were available for genome and phenotype analyses later on.

### Calculation of Generation Times

Unlike in free-living bacteria where the generation times can easily be established, this is not as straightforward for obligate intracellular bacteria since they are confined within their host. We needed to account for two aspects to estimate bacterial generation times—(i) *vertical transmission* in the host; and (ii) *horizontal transmission* through the release of EBs from the host and infection of a new host. The total number of bacterial generations then equals the sum of the generations in both transmission modes.

To determine this, we first calculated amoeba host generation numbers. Since we performed weekly transfers, the number of generations of amoebae per week was calculated by counting aliquots of amoebae in a Neubauer chamber from a particular well in the 24-well plate at the start and at the end of the week. The number of generations was then calculated using the formula *n* = log (*N*)−log (*N_0_*)/0.301, where *n* is the number of generations, *N_0_* is the initial number of amoebae and *N* is the final number of amoebae after a week. The temperature had no significant effect on the amoeba generation times, most likely due to the finite number of amoebae a well can contain. The mean number of generations per week was calculated to be 3.2. These values equate to the vertical transmission of the bacteria. Owing to the fact that the amoebae are fully infected in a continuous culture when the host cell divides, the bacteria inside will roughly divide between the two daughter cells, with each bacterium needing to divide once so as to reach the maximum bacterial number possible in a fully infected amoeba. Thus, one can assume that the amoeba generation number is equal to the bacteria generation number if one considers solely vertical transmission.

So as to assess the extent of horizontal transmission of the bacteria, it was also required to measure the amount of extracellular EBs in the host medium after one week. Briefly, this was done as follows. Culture supernatant was filtered through 1.2 µm syringe filters (Sartorius, Göttingen, Germany) to remove residual host cells. Purified bacterial EBs were then collected by centrifugation (10,000 rpm, 10 min, 4 °C), resuspended in precooled sucrose phosphate glutamate (SPG) buffer (75 g/L sucrose, 0.52 g/L KH_2_PO_4_, 1.53 g/L NaHPO_4_·7H_2_O, 1.53 g/L Na_2_HPO_4_·2H_2_O, 0.75 g/L glutamic acid; pH 7.2), homogenized using a 21-gage injection needle (B. Braun, Melsungen, Germany), and stored at −80 °C in SPG buffer. For quantification of purified EBs, 10 µL cell suspensions in 10 mL Page's amoeba saline (PAS) (0.12 g/L NaCl, 0.004 g/L MgSO_4_·7H_2_O, 0.004 g/L CaCl_2_·2H_2_O, 0.142 g/L Na_2_HPO_4_, 0.136 g/L KH_2_PO_4_) were filtered onto a polycarbonate membrane with a pore size of 0.2 µm (EMD Millipore, Billerica, MA, USA); cells were stained with 10 µg/mL 4′,6′-diamidino-2-phenylindole (DAPI) and counted using an epifluorescence microscope (Axioplan 2 imaging; Carl Zeiss, Oberkochen, Germany). The mean number of extracellular EBs released per amoeba per week was 16.

Fluorescence in situ hybridization (FISH) using a Cy3-labeled probe (E25-454, sequence GGA TGT TAG CCA GCT CAT; Thermo Fisher Scientific, Waltham, MA, USA) was then performed as described elsewhere (Wagner et al. 2003; Poppert et al. 2002; Sixt et al. 2013) so as to count, on average, how many bacterial particles were present in a fully infected amoeba, which was ∼109. Using the number of isolated EBs, this equated to an extra 0.15 generations per week (16 divided by 109). These results imply that the main mode of bacterial transmission in our experimental setup is vertical and not horizontal, with the final numbers being: 3.35 generations per weekly transfer.

### Infectivity Assays

*Protochlamydia* EBs were freshly purified from *Acanthamoeba* cultures grown in 175 cm^2^ culture flasks, in which EBs had been allowed to accumulate in TSY for 1 week. Purification of EBs was conducted as described above. Symbiont-free amoebae were harvested right before infection and an aliquot was counted in a Neubauer chamber. The amoeba cell suspension in TSY was seeded in the required amount of wells (1 mL/well) in a 24-well plate at a concentration of 10^5^ amoebae/mL. The plate was then incubated at the assay temperature (20 °C or 30 °C) for 1 h to allow the amoebae to attach before infection. EBs for the samples to be tested were thawed at 37 °C in a water bath and added in triplicate to wells in the 24-well plate at a multiplicity of infection (MOI) of 30, as this was predetermined to be the best MOI to observe differences in infectivity between samples. The well-plates were then incubated for 15 min at the assay temperature, followed by centrifugation (1,000 rpm, 15 min) at the assay temperature so as to synchronize the infection. The medium was then changed so as to remove any residual EBs from the medium. This was done by gently removing 1 mL TSY per well by pipetting, and then re-adding fresh 1 mL TSY. Wells were then sampled at 96 hpi, marking the completion of one developmental cycle. One of the wells with amoebae was treated the same way as the rest but left uninfected, and this served as a negative control to ensure that there was no infection before incubation.

FISH using a Cy3-labeled probe (E25-454, sequence GGA TGT TAG CCA GCT CAT; Thermo Fisher Scientific, Waltham, MA, USA) was then performed as described elsewhere (Wagner et al. 2003; Poppert et al. 2002; Sixt et al. 2013) on the sampled time point, and cells were also stained with 1 µg/mL DAPI for 3 min. Using an epifluorescence microscope, the number of infectious particles in 50 amoebae from each sample was counted and placed into one of three categories: uninfected, slightly infected (1–30 particles), and highly infected (>30 particles), and this allowed for the infectivity of that particular sample from the assay to be established.

Curing amoeba cultures from infection was performed in 25 cm^2^ culture flasks by adding the antibiotic Rifampicin (Sigma-Aldrich Handels GmbH, Vienna, Austria). Initially 6 µL of 100 mg/mL Rifampicin solution in DMSO was added to 10 mL TSY for a final concentration of 0.06 mg/mL Rifampicin solution. For monitoring the antibiotic effect, the infection was observed by performing FISH weekly. If the amoebae were still infected, another dose of Rifampicin solution was added to the culture and, if necessary, the dose was increased up to 12 µL of the 100 mg/mL Rifampicin solution in 10 mL TSY for a final concentration of 0.12 mg/mL Rifampicin solution. The amoeba culture was considered cured when no symbionts could be observed by FISH using the symbiont-specific probe.

### DNA Extraction and Sequencing

Frozen amoeba-chlamydia cultures were thawed in a water bath at 35 °C for 2 min and then pipetted into 25 cm^2^ culture flasks (Nalge Nunc International, Rochester, NY, USA) with 10 mL TSY to allow sufficient growth. After one week, these cultures were then upscaled into 175 cm^2^ flasks (Biologix Group Ltd, Shandong, China) for another week so as to increase the quantity of EBs that could be isolated. EB purification from the supernatant was done as described above. Isolation of genomic DNA was then carried out on these isolated EBs using a cetyl trimethylammonium bromide-based extraction method (Schmitz-Esser et al. 2010). Library preparation and pool sequencing of symbiont populations were performed at the Vienna BioCenter Core Facilities (VBCF) Next-Generation Sequencing (NGS) Unit (https://www.viennabiocenter.org/facilities/). Illumina Genome Analyser and HiSeq2000 instruments were used to generate paired-end reads of ∼125 bases according to standard procedures, achieving the desirable coverage of 300- to 600-fold.

### Sequence Read Processing and Mapping

The quality filtering and trimming of the Illumina reads was performed using Prinseq-lite (v0.19.5) (Schmieder and Edwards 2011) and Trimmomatic (v0.32) (Bolger et al. 2014) and briefly implemented as follows. Initially, a sliding window of size ten eliminated any bases with lower quality than 20 starting from the 3′ sides by removing the reading part with the low-quality bases. Any reads shorter than 40 nucleotides were also discarded. We removed any low-quality read that had an average Phred score lower than 30. Only read pairs were retained. These reads were mapped against the reference genome sequence of *P. amoebophila* UWE25 (Horn et al. 2004) (NCBI RefSeq accession number NC_005861.2) using the Burrows-Wheeler Aligner (BWA) (v0.7.5a) (Li and Durbin 2009) with standard settings and stored as bam files. For conversions from sam to bam files and from bam to fastq files (as Cortex_var input), we made use of SAMtools (v0.1.18) (Li et al. 2009) and Picard Tools (v1.92, http://broadinstitute.github.io/picard/).

### Variant Detection

We used the VarCap (v3.0) pipeline (https://github.com/ma2o/VarCap) (Zojer et al. 2017) to detect single-nucleotide and structural differences at high resolution and accuracy based on Illumina reads mapped to the ancestral genome sequence of *P. amoebophila*. VarCap is able to detect all relevant prokaryote genome variant types simultaneously with high accuracy. VarCap automatically performs quality filtering, mapping, variant calling, and postfiltering of the predicted variants.

Owing to the different variant calling abilities of the separate tools at low frequencies, various tools were combined so as to increase the sensitivity of the VarCap pipeline (Varscan2, LoFreq-Star, Pindel, Breakdancer, Delly, Cortex_var). Furthermore, a variant call had to be supported by at least two different tools so as to gain precision and robustness. Combining all these selected software tools, all variants except inversions were able to be detected at a minimum read abundance of 2% at 400 × total coverage with a minimum of 8 reads per variant, with high sensitivity. Repetitive regions that were larger than the insert size were flagged in order to mark any variants that appeared within these regions for further inspection. This was done so as to avoid false positives (FP) due to reads mapped to repetitive regions. Apart from this, to resolve FP that resulted from the incomplete detection of the true variant type, larger variants were prioritized over smaller ones. Thus, smaller variants were assigned to larger ones when they described a component of the entire variation, such as a large indel at the excision site of translocation.

The variant calling workflow was tested and evaluated using different reference bacterial and archaeal genomes, including *P. amoebophila* (Zojer et al. 2017). Low abundance (2%) variants were detected, with three being confirmed by polymerase chain reaction and Sanger sequencing. VarCap is therefore an ideal pipeline for our purpose and was selected not only due to its thorough evaluation but especially because it already had demonstrated its suitability at detecting variants in our setup.

### Estimation of Mutation Rate

In order to be able to estimate the spontaneous mutation rate, we also established three populations that were propagated for 38 months at 20 °C by monthly 1,000-fold dilutions in 1 mL TSY (allowing for ∼11 generations per month). Generation times were calculated as described above, with numbers being determined after 1 month as opposed to 1 week. MA experiments in microbes are designed in such a way as to allow mutations to arise in a neutral manner with minimal selection by repeatedly imposing tight population bottlenecks of one or a few cells for many generations (Halligan and Keightley 2009). The spontaneous mutation rate can then be estimated by simply counting the number of genetic changes that would have occurred in the evolved genome after the given amount of generations. Although we were not able to streak for single colonies on agar medium to accomplish the tight bottleneck, we consider the 1,000-fold dilution treatment at 20 °C to be somewhat of an MA experiment. The ambient temperature of the experiment imposed minimal selection on the host-symbiont system, whereas the 1,000-fold dilution reduced the effective population size drastically every month. This allowed around 11 generations of growth between every dilution, which is even fewer generations per passage than MA experiments carried out on *Salmonella typhimurium* (25 generations) and *E. coli* (28 generations) (Lind and Andersson 2008; Lee et al. 2012).

We estimated the mutation rate in two ways, one likely resulting in an overestimated value and, thus, an upper bound, and the other resulting in a much more conservative value. In both cases, we considered three replicates that were sequenced over multiple time points including the final one at 420 generations. In the first estimation, we added up all identified mutations (SNPs and small indels <4 nt) per replicate across all the sequenced time points. We then calculated the mutation rate by taking the average number of mutations per replicate and dividing by (i) genome size and total number of generations for the per nucleotide rate and; (ii) total number of generations for the per genome rate. This method clearly resulted in an exaggerated mutation rate since these mutations were identified in a population of individuals and not in single clones. Conversely, the calculations for the second conservative estimate only considered those mutations present in the final time point and whose frequency was at least 50% in the population, implying that they were present in the major clones of that specific population.

### Specificity of Genome Evolution and Affected Gene Functions

To determine the specificity of genomic evolution, we followed the calculations carried out by Deatherage and colleagues, only including those mutations that solely impacted one single gene, termed “qualifying” mutations (Deatherage et al. 2017). These included nonsynonymous SNPs as well as small insertions and deletions (<4 nt). Intergenic mutations upstream and within 250 bp of the gene start were also considered to be potential promoters affecting the nearest flanking gene (Pilalis et al. 2011). Dice's Coefficient of Similarity, *S*, was computed for each pair of evolved replicates, where *S* = 2 |*X* ∩ *Y*|/(|*X*| + |*Y*|). Here, |*X*| and |*Y*| are the sets of genes that have qualifying mutations in two replicates, and |*X* ∩ *Y*| is the set that possesses mutations in both replicates. The range of this coefficient is from 0, when the two replicates do not share any qualifying mutated genes, to 1, when both replicates have qualifying mutations in exactly the same gene set.

To investigate which gene functions were affected by mutations, genes from the final sequenced time point in which mutations were identified were assigned functional categories using protein sequences from the *P. amoebophila* genome reference through the eggNOG (v5.0) web interface. Genes with no functional prediction (i.e., no eggNOG match or assignment to categories R and S) were omitted from the analysis.

### Statistics

Statistical analysis was performed using R (v3.6.1) (R Development Core Team 2019). The statistical parameters and significance are reported in the figure legends. Data were considered to be statistically significant when *P* < 0.05. Comparisons for two groups were calculated by a two-sided Wilcoxon–Mann–Whitney test and comparisons for more than two groups were calculated by a Kruskal–Wallis test followed by Dunn's multiple comparison post-hoc test. Comparisons for paired samples were calculated by a Wilcoxon signed-rank test. The presence of a trend across multiple time points was tested using a Mann–Kendall test. One-tailed Fisher's exact tests were conducted to test whether substitutions were significantly enriched in protein-coding or intergenic regions. Gene-overlap analysis across, between, and within treatments was carried out using the R SuperExactTest package (Wang et al. 2015).

## Supplementary Material

evad139_Supplementary_Data

## Data Availability

All raw sequencing data were deposited in the National Center for Biotechnology Information (NCBI) database under bioproject accession number PRJNA723014.
