# Supplementary material for: Genome Dynamics and Temperature Adaptation During Experimental Evolution of Obligate Intracellular Bacteria
Source: Genome Biol Evol. 2023 Jul 29;15(8):evad139. doi: 10.1093/gbe/evad139 (PMC10402869; doi:10.1093/gbe/evad139)
Supplement: evad139_Supplementary_Data [file evad139_supplementary_data.zip › GBE_Herrera_Supplementary_figures.docx]

## **Genome dynamics and temperature adaptation during experimental evolution of obligate intracellular bacteria**

**Supplementary Figures**

Authors: Paul Herrera^1^*, Lisa Schuster^1^, Markus Zojer^1^, Hyunsoo Na^1,2^, Jasmin Schwarz^1^, Florian Wascher^1^, Thomas Kempinger^1^, Andreas Regner^1^, Thomas Rattei^1^, Matthias Horn^1^

Affiliations:

^1^ University of Vienna, Centre for Microbiology and Environmental Systems Science, Vienna, Austria

^2^ Current address: DOE Joint Genome Institute, Walnut Creek, CA, USA


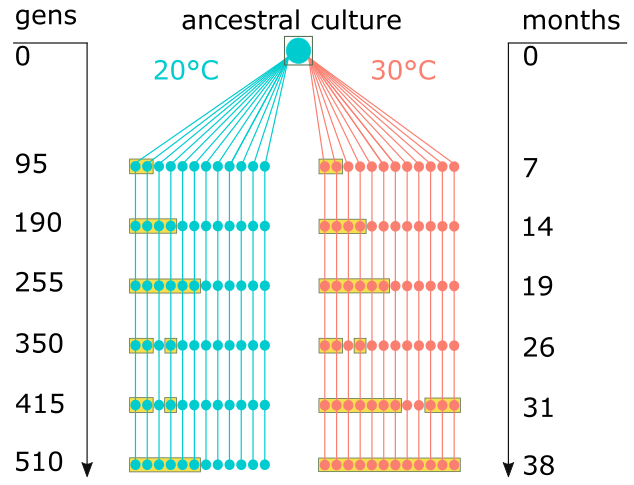


**FIG. S1. Overview of experimental setup and sequenced samples in the evolution experiment.** Pooled whole genome re-sequencing of symbiont populations was performed on numerous replicates (*n* = 61) from both treatments. Sequenced *Protochlamydia amoebophila* populations are shown in yellow boxes.


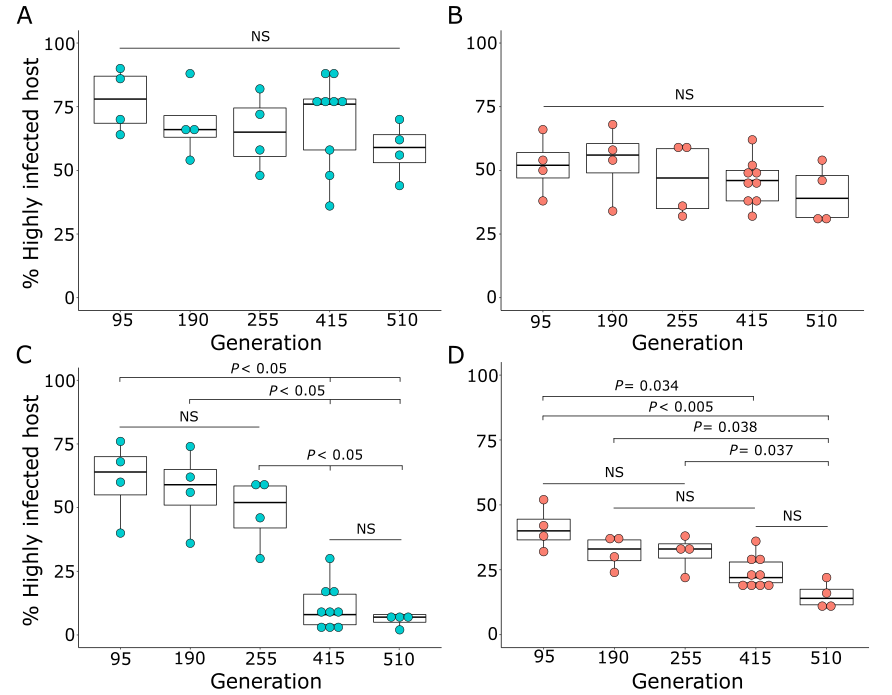


**FIG. S2. Time series analysis of symbionts isolated from the populations at both temperatures.** Naive ancestral amoebae (*n* = 4 for all generations except at 415 generations, *n* = 9) at a concentration of 10^5^ cells per ml were infected with symbionts isolated from evolved populations at different time points in the evolution experiment with a multiplicity of infection (MOI) of 30, at an assay temperature of 20 °C (blue dots) and at 30 °C (red dots). The percent of highly infected amoebae was assessed at 96 hpi using fluorescence *in situ* hybridization. In the box plots, the interquartile range (IQR) between the first and the third quartiles is indicated by the box, while vertical lines extend to a distance of 1.5 x IQR from the first or third quartile. The horizontal line within the box represents the median. **(*A* and *B*)** Infectivity assays performed using EBs isolated from the 20 °C treatment. The infectivity across the generations was compared using a Kruskal-Wallis test. **(*C* and *D*)** Infectivity assays performed using EBs isolated from the 30 °C treatment. The infectivity across the generations was compared using a Kruskal-Wallis test (C χ^2^ = 18.632, p < 0.005; D χ^2^ = 15.721, p < 0.005) followed by Dunn’s multiple comparison post-hoc test.

**
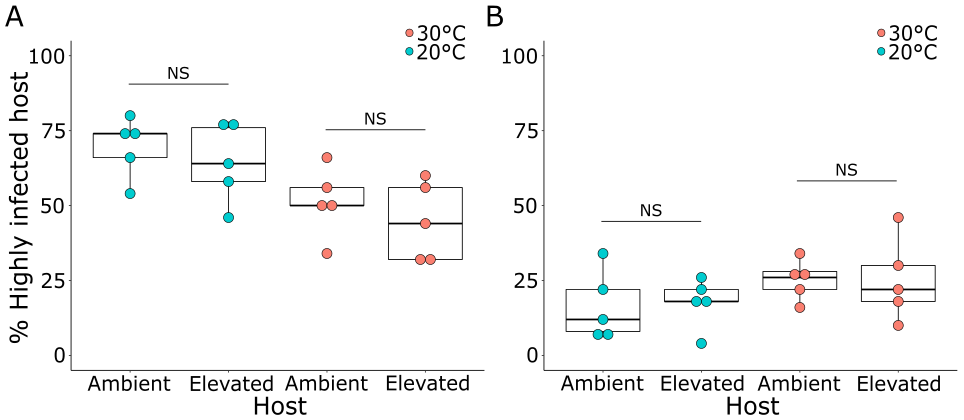
**

**FIG. S3. Infectivity of symbionts did not change when infecting hosts maintained at ambient (20 °C) or elevated (30 °C) temperature.** Amoebae maintained at 20 °C or at 30 °C (*n* = 5 for each) at a concentration of 10^5^ cells per ml were infected with symbionts isolated from evolved populations after 415 generations with an MOI of 30, at an assay temperature of 20 °C (blue dots) and at 30 °C (red dots). The percent of highly infected amoebae was assessed at 96 hpi using fluorescence *in situ* hybridization. In the box plots, the interquartile range (IQR) between the first and the third quartiles is indicated by the box, while vertical lines extend to a distance of 1.5 x IQR from the first or third quartile. The horizontal line within the box represents the median. **(*A*)** Infectivity assays performed using EBs isolated from the 20 °C treatment. The infectivity between the two hosts at the same temperature was compared using a two-sided Wilcoxon–Mann–Whitney test. **(*B*)** Infectivity assays performed using EBs isolated from the 30 °C treatment. The infectivity between the two hosts at the same temperature was compared using a two-sided Wilcoxon–Mann–Whitney test.


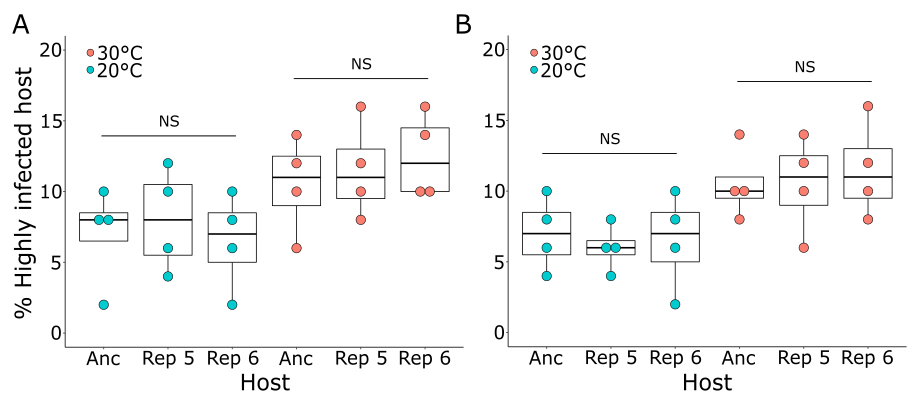


**FIG. S4. Infectivity of symbionts did not change when re-infecting their co-evolved hosts.** Naive ancestral amoebae, cured amoebae from the same population and cured amoebae from a different population (*n* = 4 for each) at a concentration of 10^5^ cells per ml were infected with symbionts isolated from two 30 °C evolved populations (replicates 5 and 6) after 510 generations with an MOI of 30, at an assay temperature of 20 °C (blue dots) and at 30 °C (red dots). The percent of highly infected amoebae was assessed at 96 hpi using fluorescence *in situ* hybridization. In the box plots, the interquartile range (IQR) between the first and the third quartiles is indicated by the box, while vertical lines extend to a distance of 1.5 x IQR from the first or third quartile. The horizontal line within the box represents the median. **(*A*)** Infectivity assays performed using EBs isolated from replicate 5. The infectivity among the host groups was compared using a Kruskal-Wallis test for each temperature. **(*B*)** Infectivity assays performed using EBs isolated from replicate 6. The infectivity among the host groups was compared using a Kruskal-Wallis test for each temperature.

**FIG. S5. Cellular functions affected by mutations.** The number of mutations assigned to cellular functions including (*A*) metabolism; (*B*) information storage and processing; and (*C*) cellular processes and signaling after 510 generations in the populations at 20 °C (blue bars) and elevated temperature (red bars) is shown. The affected functional categories differed considerably between the two temperature regimes. Mutations affected eight functional categories at 20 °C and 16 functional categories at 30 °C (supplementary table S3, Supplementary Material online).
